# Estimating risk of reported versus theoretical drug-drug interactions in headaches medicine: an exhaustive comparison between DrugBank and FAERS database for abortive and preventive combinations

**DOI:** 10.22514/jofph.2025.073

**Published:** 2025-12-12

**Authors:** Victor Kaytser, Ian Hakkinen, Jay Dave, Pengfei Zhang

**Affiliations:** ^1^UT Southwestern Medical Center, Dallas, TX 75390, USA; ^2^Evergreen Health, Seattle, WA 98272, USA; ^3^Mount Sinai Hospital, New York, NY 10029, USA; ^4^Harvard Medical School, Boston, MA 02218, USA; ^5^Department of Neurology, Beth Israel Deaconess Medical Center, Boston, MA 02218, USA

**Keywords:** Polypharmacy, Drug interactions, Combinatorics in medicine, Big data, Data science, Pharmacology

## Abstract

**Background**: Polypharmacy is common in headache medicine. This project uses DrugBank and Food and Drug Administration’s (FDA) Adverse Event Reporting System (FAERS) to determine the likelihood of a theoretical and empirically reported interaction between a given number of headache abortive and preventive medications. **Methods**: All headache medications referenced in “The American Headache Society Position Statement on Integrating New Migraine Treatments into Clinical Practice” and Szperka’s “Migraine care in the era of COVID-19: clinical pearls and plea to insurers” were included. All possible combinations of up to three abortives and preventatives were screened for drug-drug interactions through searches in DrugBank and FAERS. If at least one drug-drug interaction was listed, then it was included in our analysis. The percentage of combinations containing an interaction was then compared across the two databases. **Results**: Of the 38 abortives and 23 preventives included, once more than 3 drugs are used in any combination, a drug-drug interaction was >99% likely, per DrugBank. However, per FAERS, the reported interaction was 39% to 60% likely once 3 or 4 drugs are used in any combination. In FAERS, the likelihood of interaction rised most dramatically once 3 or more drugs are used. **Conclusions**: Theoretical interactions, based on DrugBank, maybe overstated when compared to actual observed interactions in FAERS. Future direction is needed delving into the types of interactions recorded in each database.

## 1. Introduction

Polypharmacy is common in headache medicine. The term has variable meanings in the literature, but is usually defined as the use of two or more medications in a single patient [1, 2]. Polypharmacy is often called “major” if more than four medications are involved and “minor” if otherwise [1, 2, 3]. Since headache management often requires concurrent initiation of multiple medications to stop ongoing headaches (the abortive medication) and decrease headache frequency (the preventive medication), polypharmacy is the default paradigm [1, 2, 3, 4, 5, 6, 7, 8]. In the setting of preventive medication failures, cross-tapering between medications further adds to the potential for polypharmacy [1, 9].

Although a wide range of medications have been used as abortive and preventive medications, historically, the American Headache Society (AHS) as well as the International Headache Society (IHS) have put forth lists of medications that are considered evidence-based [7, 8, 10]. Both society guidelines agree on the following classes for migraine prevention: calcitonin gene-related peptides (CGRP) receptor antagonists or antibodies, OnabotulinumtoxinA (Botox), beta-blockers, Angiotensin-Converting Enzyme (ACE) inhibitors, and anti-epileptics [7, 8]. Both guidelines also include the following list of abortive therapies: triptans, ergots, gepants, ditans, acetaminophen/paracetamol, and non-steroidal anti-inflammatory drugs (NSAIDs) [7, 10]. Finally, apart from the above, a third group contains medications used as “transition” therapy (*i.e.*, “bridging”) medications in clinical practice. The goal of transition therapy is to aggressively control headaches during special circumstances, such as status migrainosus, detoxification from medication overuse, or mini-prevention during menstrual-related migraine. One of the most well-cited lists of transition therapy is Szperka *et al*.’s [11] “Migraine care in the era of COVID-19: clinical pearls and plea to insurers”.

Drug-drug interactions (DDIs) occur when one medication influences the efficacy of another medication (pharmacodynamic interactions) or when one medication influences the absorption, metabolism, excretion, or distribution of another medication (pharmacokinetic interaction) [4, 6, 12]. DrugBank is an online database that provides comprehensive information on both types of DDIs, representing a good model for theoretical pharmacologic interactions [4, 13]. Food and Drug Administration’s (FDA) Adverse Event Reporting System (FAERS) documents medication-adverse events as well as DDIs reported to the FDA by healthcare providers, pharmaceutical companies, and consumers [5]. These interactions are by definition serious, non-trivial, or at least warrant clinician concern. The FAERS database, therefore, represents a good model for empirically reported DDIs.

Despite the prevalence of polypharmacy in headache medicine, we know of no prior study estimating the frequency of DDIs between combinations of headache abortive and preventive medications. Our project seeks to fill this gap in knowledge. Our program for investigation is as follows: (1) Using the DrugBank application programming interface (API), we seek to derive potential theoretical interactions between all possible combinations of abortives and preventives, up to three medications in each category. (2) Using FAERS API, we seek to determine the empirically observed and reported interactions between these combinations of abortive and preventive medications. (3) Finally, we compared both databases to generate a list of the most common and least common interacting medications from both a theoretical and an empirical perspective.

## 2. Materials and methods

Our study has four phases: (1) medication selection phase. (2) combinatorics phase. (3) DrugBank data access and screening phase. (4) FAERS data access and screening phase.

### 2.1 Medication selection phase

To obtain an up-to-date and well-established list of medications as input, we gathered all abortive and preventive medications referenced from 2019 AHS Consensus Statement, as well as Spzerka *et al*.’s [11] “Migraine care in the era of COVID-19: clinical pearls and plea to insurers” [14]. We made the following adjustments to the above lists: “bridge” medications are considered “abortive” medications in our project. We excluded intranasal lidocaine, as it is minimally systemically absorbed. Valproic acid and amitriptyline are cited as both preventive and abortive medications; they are considered preventive medications in our model [11, 14]. Frovatriptan is also classified as both preventive and abortive medication; we decided to label it as an abortive medication only. Combination analgesics were broken down into individual components. Neuromodulators were excluded as they are not part of the FAERS or DrugBank database. The included list of abortives and preventive medications is listed in Table 1. Brand names were standardized to generic equivalents according to **Supplementary Table 1** to ensure consistency across all phases of the study.

**Table 1.** List of headache medications included.

Abortives	Preventives
Almotriptan	Galcanezumab
Eletriptan	Erenumab
Rizatriptan	Fremanezumab
Sumatriptan	Eptinezumab
Naratriptan	OnabotulinumtoxinA
Frovatriptan	Candesartan
Zolmitriptan	Lisinopril
Ubrogepant	Melatonin
Rimegepant	Zonisamide
Lasmiditan	Valproate
Indomethacin	Topiramate
Ketorolac	Metoprolol
Naproxen	Propranolol
Nabumetone	Timolol
Diclofenac	Amitriptyline
Prochlorperazine	Venlafaxine
Promethazine	Atenolol
Metoclopramide	Nadolol
Chlorpromazine	Clonidine
Olanzapine	Guanfacine
Quetiapine	Nebivolol
Methylprednisolone	Pindolol
Dexamethasone	Cyproheptadine
Prednisone	
Hydroxyzine	
Tizanidine	
Magnesium	
Dihydroergotamine	
Aspirin	
Ibuprofen	
Butorphanol	
Flurbiprofen	
Ergotamine	
Isometheptene	
Acetaminophen	
Codeine	
Tramadol	
Droperidol	

### 2.2 Combinatorics phase

Once the list of input medications was compiled, we algorithmically obtained all possible combinations of N many abortives against M many preventive medications, where N is in the set of {1, 2, 3} and M is in the set of {1, 2, 3}. We denoted each of these sets as abortiveNpreventiveM. For example, the set abortive3preventive2 denotes all possible combinations of 3 abortive medications and 2 preventive medications. These lists of all medication combinations up to 3 abortives and 3 preventives were then used for the following two phases.

### 2.3 DrugBank data access and screening phase

We used the following method to determine the number of theoretical interactions based on DrugBank data as a function of N and M: (1) We generated all possible combinations, when taken two at a time without repetition, for abortive medications; we denoted this list of combinations as L1. (2) We obtained a list of all possible combinations of preventive medications when taken two at a time without repetition; we denoted this list as L2. (3) Finally, we generated a list of all combinations between 1 abortive and 1 preventive medication; we denoted this list as L3.

We then used DrugBank API to determine whether any interactions exist between each of the elements of L1, L2, and L3. The resulting lists, L1*, L2*, L3* represent all potential drug-drug interactions between 2 abortives, between 2 preventives, as well as between 1 abortive and 1 preventive, respectively.

Using L1*, L2*, L3*, we then screened all combinations of N abortives and M preventives for interactions (If an interaction exists in abortiveNpreventiveM, then it was an interaction denoted in L1*, L2*, and L3*). In other words, each item in each set of the abortiveNpreventiveM medications was screened for potential interactions based on L1*, L2*, and L3* results. We denoted the result abortiveNpreventiveM*. These values denoted the theoretical interactions between all possible combinations of medications up to 3 abortives and 3 preventives.

### 2.4 FAERS data access and screening phase

For the FAERS portion of the study, we downloaded and parsed all XML files from the FDA website from October 2012 to March 2020. All major brand names were converted into generics in the database (see **Supplementary Table 1** for complete mapping). We screened for all specific interactions that implicated two or more of our abortive and/or preventive medications. We denoted the resultant list as F.

List F was then used to filter out all N abortives and M preventives combinations that contain any interacting medications. That is, if any element is a subset of a drug combination that is in abortiveNpreventiveM, then it was tagged as a drug-drug interaction. The resultant list would be denoted as abortiveNpreventiveM**. These values denoted the reported interactions between all possible combinations of medications up to 3 abortives and 3 preventives. Standardized generics ensured that reported interactions, such as “Relpax” or “Zomig”, were properly matched to their respective generic forms (*e.g.*, eletriptan, zolmitriptan; **Supplementary Table 1**).

Manual verification of data suggested that DrugBank’s description of candesartan’s interactions with other preventive medications is incomplete/erroneous. A manual verification of interactions for candesartan was performed.

All utilization of API was accessed through the Python programming language. All data manipulation, including text processing, was accomplished through Haskell programming language. All programming sources are proprietary and composed by the authors (Selected source codes are available by request).

## 3. Results

DrugBank and FAERS data were downloaded on 26 August 2020. Mefenamic acid was not included in DrugBank for interaction at the time of download and was therefore excluded. Our data contained 38 abortive medications and 23 preventive medications. This list of inclusion medications is displayed in Table 1. All brand names were standardized to generic equivalents using **Supplementary Table 1**, ensuring consistency across DrugBank and FAERS sources.

Table 2 displays the total number of possible combinations of abortive and preventive medications, as well as the number of interactions between various combinations of abortives and preventives from DrugBank and FAERS data. We were unable to complete 3 abortive medications against 3 preventive medications (*i.e.*, 14,940,156 combinations, or _38_C_3_ multiplied by _23_C_3_) due to limitations in our computational hardware. For a summary of how these combinations were generated and screened for interactions, see the four-phase methodology diagram (Fig. 1).

**Table 2.** Observed interactions based on the number of abortive/preventive medications used in DrugBank and FAERS.

DrugBank				FAERS			
Abortives	Preventives			Abortives	Preventives		
	1	2	3		1	2	3
Number of Interactions							
1	628	9146	66,994	1	151	3725	39,584
2	15,350	177,289	1,244,829	2	6392	103,891	915,594
3	193,185	2,134,216	*	3	115,799	1,574,622	*
Number of Possible Combinations							
1	874	9614	67,298	1	874	9614	67,298
2	16,169	177,859	1,245,013	2	16,169	177,859	1,245,013
3	194,028	2,134,308	*	3	194,028	2,134,308	*
Probability of Interaction							
1	0.7185354	0.9513209	0.9954827	1	0.1727688	0.3874557	0.5881898
2	0.9493475	0.9967952	0.9998522	2	0.3953243	0.5841200	0.7354091
3	0.9956552	0.9999568	*	3	0.5968159	0.7377669	*

*: Not calculated. FAERS: FDA’s Adverse Event Reporting System.

**Fig. 1.** Diagram of study protocol. FAERS: FDA’s Adverse Event Reporting System.

We counted the occurrences when each medication came up as interacting for specific combinations of medications. We presented the top 10 least-interacting medications for 1 abortive *vs.* 1 abortive, 1 abortive *vs.* 1 preventive, and 1 preventive *vs.* 1 preventive for each database, in Tables 3 and 4, respectively. The complete set of this data is in **Supplementary Tables 2 and 3**, respectively. For example, when all possible combinations of two abortive medications are combined, ubrogepant occurred 5 times as interacting according to DrugBank, but 0 times in the FAERS database (since it is not listed in column 1 of Table 4). All interactions reference generic drug names.

**Table 3.** Frequency of interactions between medications in DrugBank (top 10 least interacting).

Drugbank as histogram		
Abortive2preventive0	Abortive0preventive2	Abortive1preventive1
Ubrogepant 5	Eptinezumab 3	Eptinezumab 1
Rimegepant 7	Erenumab 3	Erenumab 1
Magnesium 13	Fremanezumab 3	Fremanezumab 1
Methylprednisolone 17	Galcanezumab 3	Galcanezumab 1
Prednisone 21	Onabotulinum toxin 10	Methylprednisolone 2
Butorphanol 23	Melatonin 11	Rimegepant 4
Hydroxyzine 23	Cyproheptadine 12	Ubrogepant 4
Prochlorperazine 24	Atenolol 13	Magnesium 6
Acetaminophen 24	Candesartan 13	Butorphanol 12
Lasmiditan 24	Lisinopril 14	Prednisone 13

**Table 4.** Frequency of interactions between medications in FAERS (top 10 least interacting).

FAERS data as histogram		
Abortive2preventive0	Abortive0preventive2	Abortive1preventive1
Eletriptan 1	Erenumab 1	Eletriptan 1
Nabumetone 1	Fremanezumab 1	Erenumab 1
Naratriptan 1	Galcanezumab 1	Ergotamine 1
Rizatriptan 1	Guanfacine 1	Frovatriptan 1
Zolmitriptan 1	Cyproheptadine 2	Galcanezumab 1
Prochlorperazine 3	Nebivolol 2	Guanfacine 1
Flurbiprofen 3	Timolol 2	Ketorolac 1
Tizanidine 3	Zonisamide 2	Naratriptan 1
Droperidol 5	Melatonin 3	Fremanezumab 2
Chlorpromazine 6	Atenolol 4	OnabotulinumtoxinA 2

*FAERS: FDA’s Adverse Event Reporting System.*

### 3.1 DrugBank results

As expected, increased combinations of abortive and preventive medications result in increased drug-drug interactions. Seventy-two percent (71.85%) of combinations between 1 abortive and 1 preventive were noted as interacting. When four drugs are in combination (N = 2, M = 2; or N = 1, M = 3; or N = 3, M = 1), the probability of drug-drug interactions between the headache medications is >95% (Table 2, Fig. 2). When any abortive and preventive medication combinations (N greater or equal to 1 and M greater or equal to 1) were picked, the probability of an interaction based on DrugBank is greater than 70%.

**Fig. 2.** Heatmaps of drug interactions probability across drug combinations. FAERS: FDA’s Adverse Event Reporting System.

Ubrogepant, rimegepant, magnesium, methylprednisolone, and prednisone are least interacting when combining two abortive medications. Whereas combining dexamethasone, ergotamine, chlorpromazine, tramadol, and quetiapine are most interacting. When combining two preventative medications, eptinezumab, erenumab, fremanezumab, galcanezumab, and onabotulinumtoxinA are least interacting, while zonisamide, pindolol, guanfacine, clonidine, and amitriptyline are the most interacting. Finally, when combining one abortive with one preventive, eptinezumab, erenumab, fremanezumab, galcanezumab, and methylprednisolone are least interacting. Candesartan, zonisamide, venlafaxine, topiramate, amitriptyline are most interacting (See Table 3). See Fig. 1 for a visual reference of how DrugBank interactions (L1, L2, L3*) were applied during screening.

### 3.2 FAERS results

When one abortive and one preventive medications are combined, 17.27% of combinations contain an interaction. When four drugs are in combination (N = 2, M = 2; or N = 1, M = 3; or N = 3, M = 1), the probability of an interaction is between 58% and 60% (Table 2). When less than four medications are used in combination, the probability of an interaction is less than 40% (Fig. 2). All drug names were normalized to their generic equivalents using Table 2 prior to the filtering for known interactions.

Based on FAERS data, almotriptan, frovatriptan, ubrogepant, rimegepant, lasmiditan, butorphanol, ergot/dihydroergotamine (DHE), and isometheptene are least interacting when combining two abortive medications, while tramadol, ibuprofen, diclofenac, quetiapine, and acetaminophen are most interacting. When combining two preventative medications, onabotulinum toxin, nadolol, pindolol, erenumab, fremanezumab, galcanezumab, guanfacine are least interacting. Venlafaxine, valproate, candesartan, propranolol, metoprolol are the most interacting. See Fig. 1 for how FAERS-reported interactions (List F) were filtered against all drug combinations (abortiveNpreventiveM) to yield final results. Finally, when combining one abortive with one preventive, eletriptan, erenumab, ergotamine, frovatriptan, galcanezumab are least interacting. Venlafaxine, valproate, amitriptyline, lisinopril, metoprolol are most interacting (See Table 3 and **Supplementary Table 3**).

## 4. Discussion

Our study of DDIs in a “combinatoric” fashion is the third of its kind following previous studies on abortive and preventive medication combinations [4, 6]. This paper addresses the limitation to our prior methodology by: (1) including interactions between abortive and preventive medications to model realistic clinical scenarios; and (2) including the FAERS database, which allows for an estimate of empirical DDIs. These modifications challenge our prior conclusions: observed, or at least reported, interactions are likely to be significantly lower in real-life settings than is suggested by DrugBank. In this study, any three-drug combination, whether the drug is preventive or abortive, contains at least one interaction in 94% to 95% of combinations in DrugBank, but only 38% to 39% of combinations in FAERS contain an interaction. Any four-drug combination appears to produce a theoretical risk of 99% probability of interaction per DrugBank, but was observed to produce less than 59% interaction in FAERS. Finally, according to FAERS, a probability of interaction greater than 70% only occurs if five-drug combinations are used. In other words, there is greater than 60% chance of no interactions as long as fewer than 4 medications are used in combination. Therefore, clinicians can be assured that the risk of a drug-drug interaction is less than 60% when no more than three drugs are prescribed in combination.

For abortive medications, ubrogepant and rimegepant appear to be the least interacting in both DrugBank and FAERS. For preventive medications, CGRP monoclonal antibodies and Botox are the least interacting. It is no surprise that onabotulinumtoxinA is the least interacting, given that it is only minimally systemically distributed [15]. The inclusion of “gepants” and monoclonal antibodies appears to be supported by evidence external to our projects [16, 17, 18, 19, 20, 21, 22]. Of course, we cannot discount the argument that these novel therapies simply have not been tested long enough in the real-world setting for interactions to be revealed; CGRP monoclonal antibodies were FDA approved in May 2018, whereas “gepants” were approved starting in December of 2019. Therefore, our FAERS analysis would only include approximately 22 months of data for the former and 4 months of data for the latter.

In FAERS, almotriptan, frovatriptan, and eletriptan are also the least interacting. This should not be surprising to headache clinicians; despite the “triptan-phobia” associated with discredited theoretical interactions such as serotonin syndrome, clinical data have consistently shown that triptans are usually well tolerated [23, 24, 25]. Our data appears to support this claim.

Tramadol and quetiapine are among the top interacting abortive medications in both databases. This highlights the risks involved in using opioids for abortive therapy in patients with polypharmacy. For preventative medications, venlafaxine and amitriptyline are amongst the most interacting. Amitriptyline is, of course, one of the evidence-based medications against episodic migraine and is often the first-line medication for migraine prevention [14]. Its inclusion as the most interacting should encourage clinicians to discontinue the medication once its failure has been established in a preventative medication trial. Alternatively, if polypharmacy were necessary, using amitriptyline in conjunction with less interacting preventive medications, such as onabotulinumtoxinA or CGRP monoclonal antibodies, would be prudent. Similar statements can be made for venlafaxine. Since venlafaxine can be used for mood disorders and chronic pain, two of the most common comorbidities in headache patients, vigilance to drug-drug interaction is strongly advised when adding a headache-preventive medication.

A few medications that offer seemingly conflicting results between the two methods demonstrate the limitations of the two databases. In other words, when a medication is the most interacting in one database but least interacting in another, we take this polarization as reflecting the idiosyncrasies of the databases. For example, steroids are both the most interacting and the least interacting in DrugBank. This question was addressed in our prior pilot study and is one of the weaknesses of DrugBank; the paradox was ascribed to theoretical idiosyncrasies of steroids interactions with acetaminophen [4]. As expected, this phenomenon was not observed with FAERS. Ergotamine is the most interacting medication in DrugBank for two-abortive combinations, but the least interacting in FAERS in the same category. We suspect that this is due to the infrequent use of ergotamine compared to other medications. Similarly, pindolol and guanfacine are the most interacting medications in the two-preventive categories in DrugBank, but the least interacting in FAERS. We suspect that this is because neither drug is a popular pharmaceutical choice, as evidenced by prescription sale data [26, 27].

In summary, this paper’s selection of medications offers support for the use of CGRP antagonists as well as onabotulinumtoxinA for the prevention of migraine. We must note, however, that the former medications are “younger” as compared with other preventive medications; while we are optimistic regarding the safety profiles for these medications, we caution clinicians to remain vigilant to their potential or yet-undiscovered side effects.

Empirically, clinicians should attempt to keep the total number of headache medications to fewer than four; any combination of abortive/prevention combination exceeding four is more likely than not to cause some sort of interaction. Since severity of side effects is not documented in FAERS, we are not advocating clinicians to completely avoid four drug combinations; rather, we advise careful monitoring in such circumstances.

Although outside the scope of this paper, clinicians should also take into account the interactions between non-headache medications and headache medications. For example, caution should be taken for the use of topiramate in patients using oral contraceptives due to the medications’ known interactions [28]. Candesartan should not be used with spironolactone, which is commonly used for the treatment of acne [29]. Antidepressants for migraine prevention should be used judiciously in those already taking another antidepressant, due to the potential for serotonin syndrome. On the abortive side, serotonergic medications should be avoided in patients using linezolid [30].

An important limitation in our study is that our methods allow for any single report of an adverse event in FAERS to count towards the adverse event probability. Therefore, as a conservative way of accounting for adverse events, the number of reported interactions in our data should not be construed as a “true” estimate but rather an upper bound for real-life adverse events. Furthermore, for individual patients, the probability of a drug-drug interaction is likely somewhere between the theoretical and the empirical probabilities, given idiosyncratic metabolic and genetics profiles.

DrugBank and FAERS also do not rank clinical severity of potential or reported medication-adverse events. As a result, a limitation to our study is that this information is not available to inform our research. In other words, minor adverse events are counted similarly to severe ones. A future direction would be to incorporate this into our research. The challenge here would be to “rank” adverse events in terms of severity, requiring the translation of a multifaceted phenomenon into a linear/numerical one for the purpose of ranking. One way of doing so may be through the calculation of disability-adjusted life years as a measurement. This may facilitate clinicians in making rational risk/benefit decisions when choosing a headache regimen for their patients.

Finally, there are limitations inherit to using FAERS for our study. When the FAERS dataset tags a complaint as the result of drug-drug interaction, the offending medications are tagged in the dataset. However, it is possible for a complaint to tag multiple medications as interacting when they are not, in fact, doing so. For example, case 9848824 tags “hyponatremia” and “c-reactive protein increased” as a drug interaction between tegretol, valproic acid, lamotrigine, hydrocortisone, and fludrocortisone acetate. In this case, whether fludrocortisone and hydrocortisone are contributing to the majority of the interaction is unclear.

DrugBank and FAERS each contain inherent limitations and biases. Our goal in this study is not to reconcile some of these immutable limitations in each database, but rather to look at both databases concurrently. Thus, balancing the weakness in one database against the strength of the other produces an estimate of drug-drug interactions in real-life settings.

## 5. Conclusions

Theoretical interactions between migraine medications may be overstated when compared to actual observed interactions. CGRP antagonists and Botox appear to be the least-interacting preventative medications, whereas triptans and gepants appear to be the least-interacting abortive medications.

## Supplementary Material

Supplementary material associated with this article can be found, in the online version, at https://files.jofph.com/ files/article/1999369037891420160/attachment/ Supplementary-material.docx.


