# Supplementary material for: Estimating risk of reported versus theoretical drug-drug interactions in headaches medicine: an exhaustive comparison between DrugBank and FAERS database for abortive and preventive combinations
Source: J Oral Facial Pain Headache. 2025 Dec 12;39(4):165–72. doi: 10.22514/jofph.2025.073 (PMC12727184; doi:10.22514/jofph.2025.073)
Supplement: Supplementary file 1 [file Supplementary-material.docx]

Supplementary material

Supplementary Table 1. Generics name their equivalent in FAERS.

| Generic | Brand name |
| --- | --- |
| Almotriptan | Almotriptan, axert |
| Eletriptan | Eletriptan, relpax |
| Rizatriptan | Rizatriptan, maxalt |
| Sumatriptan | Sumatriptan, imitrex, onzentra, zembrace, sumavel, alsuma, migranow, tosymra |
| Naratriptan | Naratriptan, nara |
| Frovatriptan | Frovatriptan, frova |
| Zolmitriptan | Zolmitriptan, zomig |
| Ubrogepant | Ubrogepant, ubrelvy |
| Rimegepant | Rimegepant, nurtec |
| Lasmiditan | Lasmiditan, reyvow |
| Indomethacin | Indomethacin, indocin |
| Ketorolac | Ketorolac, toradol |
| Naproxen | Naproxen, anaprox, naprelan, naprosyn, aleve, mediproxen |
| Nabumetone | Nabumetone, relafen |
| Diclofenac | Diclofenac, voltaren, cataflam, cambia |
| Mefenamic | Mefenamic, ponstel |
| Prochlorperazine | Prochlorperazine, chlormeprazine, chloropernazine, compazine |
| Promethazine | Promethazine, phenergan, phenadoz, promethegan |
| Metoclopramide | Metoclopramide, metozolv, octamide |
| Chlorpromazine | Clomipramine, thorazine |
| Olanzapine | Olanzapine, zyprexa, zydis, relprevv |
| Quetiapine | Quetiapine, seroquel |
| Valproic acid | Valproate, depakote, depakene, depacon, stavzor, divalproex, valproic acid |
| Methylprednisolone | Methylprednisolone, medrol, depo-medrol, solu-medrol, a-methapred |
| Dexamethasone | Dexamethasone, decadron, dexpak |
| Prednisone | Prednisone, deltasone |
| Hydroxyzine | Hydroxyzine, atarax, vistaril |
| Amitriptyline | Amitriptyline, elavil, endep |
| Tizanidine | Tizanidine, zanaflex |
| Magnesium | Magnesium |
| Dihydroergotamine | Dihydroergotamine, DHE, migranal |
| Aspirin | Aspirin, bayer, asatab, ascriptin, aspirtab, ecotrin, entercote |
| Ibuprofen | Ibuprofen, advil, motrin, medipren, nuprin |
| Butorphanol | Butorphanol, stadol |
| Flurbiprofen | Flurbiprofen, ansaid, ocufen, strepfen |
| Ergotamine | Ergotamine, cafergot, ergomar |
| Isometheptine | Isometheptine, isometheptine |
| Acetaminophen | Acetaminophen, paracetamol, tylenol |
| Codeine | Codeine |
| Tramadol | Tramadol, ultram, conzip |
| Droperidol | Droperidol, inapsine |
| Galcanezumab | Galcanezumab, emgality |
| Erenumab | Erenumab, aimovig |
| Fremanezumab | Fremanezumab, ajovy |
| Eptinezumab | Eptinezumab, vyepti |
| OnabotulinumtoxinA | OnabotulinumtoxinA, botox |
| Candesartan | Candesartan, atacand |
| Lisinopril | Lisinopril, zestril, prinvil, qbrelis |
| Melatonin | Melatonin |
| Zonisamide | Zonisamide, zonegran |
| Metoprolol | Metoprolol, lopressor, toprol |
| Propranolol | Propranolol, inderal, innopran, hemangeol |
| Timolol | Timolol, betimol, istalol |
| Venlafaxine | Venlafaxine, effexor |
| Atenolol | Atenolol, tenormin |
| Nadolol | Nadolol, corgard |
| Clonidine | Clonidine, catapres |
| Guanfacine | Guanfacine, intuniv, tenex |
| Nebivolol | Nebivolol, bystolic, byvalson |
| Pindolol | Pindolol, visken |
| Cyproheptadine | Cyproheptadine, periactin |
| Topiramate | Topiramate, topamax, trokendi, qudexy, topiragen |

DHE: dihydroergotamine.

Supplementary Table 2. Frequency of interactions between medications in DrugBank.

| Drugbank as histogram | | |
| --- | --- | --- |
| Abortive2preventive0 | Abortive0preventive2 | Abortive1preventive1 |
| Ubrogepant, 5 | Eptinezumab, 3 | Eptinezumab, 1 |
| Rimegepant, 7 | Erenumab, 3 | Erenumab, 1 |
| Magnesium, 13 | Fremanezumab, 3 | Fremanezumab, 1 |
| Methylprednisolone, 17 | Galcanezumab, 3 | Galcanezumab, 1 |
| Prednisone, 21 | Onabotulinum toxin, 10 | Methylprednisolone, 2 |
| Butorphanol, 23 | Melatonin, 11 | Rimegepant, 4 |
| Hydroxyzine, 23 | Cyproheptadine, 12 | Ubrogepant, 4 |
| Prochlorperazine, 24 | Atenolol, 13 | Magnesium, 6 |
| Acetaminophen, 24 | Candesartan, 13 | Butorphanol, 12 |
| Lasmiditan, 24 | Lisinopril, 14 | Prednisone, 13 |
| Aspirin, 27 | Nadolol, 14 | Isometheptene, 15 |
| Isometheptene, 27 | Metoprolol, 15 | Acetaminophen, 17 |
| Ibuprofen, 28 | Nebivolol, 15 | Aspirin, 17 |
| Tizanidine, 28 | Valproate, 15 | Diclofenac, 17 |
| Flurbiprofen, 29 | Timolol, 16 | Flurbiprofen, 17 |
| Ketorolac, 29 | Topiramate, 16 | Ibuprofen, 17 |
| Naproxen, 29 | Propranolol, 17 | Indomethacin, 17 |
| Codeine, 30 | Venlafaxine, 17 | Ketorolac, 17 |
| Diclofenac, 30 | Amitriptyline, 18 | Nabumetone, 17 |
| Indomethacin, 30 | Clonidine, 18 | Naproxen, 17 |
| Nabumetone, 30 | Guanfacine, 18 | Prochlorperazine, 18 |
| Dihydroergotamine, 31 | Pindolol, 18 | Hydroxyzine, 18 |
| Droperidol, 31 | Zonisamide, 18 | Lasmiditan, 18 |
| Naratriptan, 31 |  | Almotriptan, 19 |
| Frovatriptan, 32 |  | Chlorpromazine, 19 |
| Zolmitriptan, 32 |  | Codeine, 19 |
| Almotriptan, 33 |  | Dihydroergotamine, 19 |
| Eletriptan, 33 |  | Droperidol, 19 |
| Olanzapine, 33 |  | Eletriptan, 19 |
| Promethazine, 33 |  | Ergotamine, 19 |
| Rizatriptan, 33 |  | Frovatriptan, 19 |
| Sumatriptan, 33 |  | Metoclopramide, 19 |
| Metoclopramide, 34 |  | Naratriptan, 19 |
| Quetiapine, 34 |  | Olanzapine, 19 |
| Tramadol, 34 |  | Promethazine, 19 |
| Chlorpromazine, 35 |  | Quetiapine, 19 |
| Ergotamine, 35 |  | Rizatriptan, 19 |
| Dexamethasone, 37 |  | Sumatriptan, 19 |
|  |  | Tizanidine, 19 |
|  |  | Tramadol, 19 |
|  |  | Zolmitriptan, 19 |
|  |  | Dexamethasone, 23 |
|  |  | Onabotulinum toxin, 24 |
|  |  | Cyproheptadine, 25 |
|  |  | Lisinopril, 30 |
|  |  | Atenolol, 32 |
|  |  | Melatonin, 32 |
|  |  | Clonidine, 33 |
|  |  | Nadolol, 33 |
|  |  | Nebivolol, 33 |
|  |  | Propranolol, 33 |
|  |  | Timolol, 33 |
|  |  | Guanfacine, 34 |
|  |  | Metoprolol, 34 |
|  |  | Pindolol, 34 |
|  |  | Valproate, 34 |
|  |  | Amitriptyline, 35 |
|  |  | Topiramate, 35 |
|  |  | Venlafaxine, 36 |
|  |  | Zonisamide, 36 |
|  |  | Candesartan, 38 |

Supplementary Table 3. Frequency of interactions between medications in FAERS.

| FAERS data as histogram | | |
| --- | --- | --- |
| Abortive2preventive0 | Abortive0preventive2 | Abortive1preventive1 |
| Eletriptan, 1 | Erenumab, 1 | Eletriptan, 1 |
| Nabumetone, 1 | Fremanezumab, 1 | Erenumab, 1 |
| Naratriptan, 1 | Galcanezumab, 1 | Ergotamine, 1 |
| Rizatriptan, 1 | Guanfacine, 1 | Frovatriptan, 1 |
| Zolmitriptan, 1 | Cyproheptadine, 2 | Galcanezumab, 1 |
| Prochlorperazine, 3 | Nebivolol, 2 | Guanfacine, 1 |
| Flurbiprofen, 3 | Timolol, 2 | Ketorolac, 1 |
| Tizanidine, 3 | Zonisamide, 2 | Naratriptan, 1 |
| Droperidol, 5 | Melatonin, 3 | Fremanezumab, 2 |
| Chlorpromazine, 6 | Atenolol, 4 | Onabotulinum toxin, 2 |
| Sumatriptan, 6 | Topiramate, 5 | Sumatriptan, 2 |
| Indomethacin, 8 | Amitriptyline, 6 | Timolol, 2 |
| Codeine, 9 | Clonidine, 6 | Tizanidine, 2 |
| Ketorolac, 9 | Lisinopril, 7 | Cyproheptadine, 3 |
| Hydroxyzine, 10 | Metoprolol, 8 | Zonisamide, 3 |
| Methylprednisolone, 10 | Propranolol, 8 | Chlorpromazine, 4 |
| Metoclopramide, 10 | Candesartan, 9 | Metoclopramide, 4 |
| Promethazine, 10 | Valproate, 10 | Promethazine, 4 |
| Dexamethasone, 11 | Venlafaxine, 10 | Dexamethasone, 5 |
| Naproxen, 12 |  | Methylprednisolone, 5 |
| Prednisone, 12 |  | Rizatriptan, 5 |
| Olanzapine, 13 |  | Melatonin, 6 |
| Magnesium, 15 |  | Nebivolol, 6 |
| Aspirin, 16 |  | Codeine, 7 |
| Acetaminophen, 17 |  | Prednisone, 7 |
| Quetiapine, 17 |  | Acetaminophen, 8 |
| Diclofenac, 18 |  | Hydroxyzine, 8 |
| Ibuprofen, 18 |  | Naproxen, 8 |
| Tramadol, 18 |  | Magnesium, 9 |
|  |  | Olanzapine, 9 |
|  |  | Topiramate, 9 |
|  |  | Atenolol, 10 |
|  |  | Candesartan, 10 |
|  |  | Clonidine, 10 |
|  |  | Diclofenac, 10 |
|  |  | Propranolol, 10 |
|  |  | Aspirin, 12 |
|  |  | Quetiapine, 12 |
|  |  | Tramadol, 12 |
|  |  | Ibuprofen, 13 |
|  |  | Metoprolol, 13 |
|  |  | Lisinopril, 14 |
|  |  | Amitriptyline, 15 |
|  |  | Valproate, 15 |
|  |  | Venlafaxine, 18 |

FAERS: FDA’s Adverse Event Reporting System.
